# Clonal expansion of SIV-infected cells in macaques on antiretroviral therapy is similar to that of HIV-infected cells in humans

**DOI:** 10.1371/journal.ppat.1007869

**Published:** 2019-07-10

**Authors:** Andrea L. Ferris, David W. Wells, Shuang Guo, Gregory Q. Del Prete, Adrienne E. Swanstrom, John M. Coffin, Xiaolin Wu, Jeffrey D. Lifson, Stephen H. Hughes

**Affiliations:** 1 HIV Dynamics and Replication Program, National Cancer Institute Frederick, National Institutes of Health, Frederick, MD, United States of America; 2 Cancer Research Technology Program, Leidos Biomedical Research Inc., Frederick National Laboratory for Cancer Research, Frederick MD, United States of America; 3 AIDS and Cancer Virus Program, Leidos Biomedical Research, Inc., Frederick National Laboratory for Cancer Research, Frederick, MD, United States of America; 4 Department of Molecular Biology and Microbiology, Tufts University, Boston MA, United States of America; Boston College, UNITED STATES

## Abstract

Clonal expansion of HIV infected cells plays an important role in the formation and persistence of the reservoir that allows the virus to persist, in DNA form, despite effective antiretroviral therapy. We used integration site analysis to ask if there is a similar clonal expansion of SIV infected cells in macaques. We show that the distribution of HIV and SIV integration sites in vitro is similar and that both viruses preferentially integrate in many of the same genes. We obtained approximately 8000 integration sites from blood samples taken from SIV-infected macaques prior to the initiation of ART, and from blood, spleen, and lymph node samples taken at necropsy. Seven clones were identified in the pre-ART samples; one persisted for a year on ART. An additional 100 clones were found only in on-ART samples; a number of these clones were found in more than one tissue. The timing and extent of clonal expansion of SIV-infected cells in macaques and HIV-infected cells in humans is quite similar. This suggests that SIV-infected macaques represent a useful model of the clonal expansion of HIV infected cells in humans that can be used to evaluate strategies intended to control or eradicate the viral reservoir.

## Author summary

Although antiretroviral therapy (ART) effectively blocks HIV replication, infected people are not cured. As a part of its normal replication cycle, HIV inserts (integrates) a DNA copy of its genome into the genome of infected host cells, which allows the virus to persist as long as the infected cells survive. Not only can these infected cells survive, they can grow and divide, increasing the numbers of infected cells without viral replication. The ability of the infected cells to proliferate plays an important role in maintaining the numbers of infected cells (and the infection) in people on successful therapy. However, there are some important experiments that cannot easily be done with samples that can be obtained from HIV infected people. SIV infected macaques are often used as a model to do experiments that cannot be done in HIV infected people. We show here that the distribution of HIV and SIV integration sites is similar, and that, in infected macaques, the timing and extent of the proliferation of SIV infected cells is also quite similar to HIV infected cells in humans. This shows that the SIV/macaque system can be used to model the clonal expansion of HIV infected cells.

## Introduction

T cells clonally expand in response to homeostatic/cytokine and antigen-specific stimuli as part of their normal physiology. Not surprisingly, some HIV-infected CD4+ T-cells also clonally expand [1, 2], and expanded clones can persist in infected individuals for more than 10 years. Analysis of clonal expansion of HIV infected cells has been done primarily using PBMCs obtained from the blood of patients who had been on suppressive combination antiviral therapy (ART) for at least several years when the samples were taken. Effective ART either greatly reduces, or entirely prevents, the de novo infection of additional T cells in HIV infected humans and RT-SHIV infected macaques [3–5]. Because ART blocks new rounds of HIV infection, and because most infected T cells die quickly after infection, clones are more easily detected in individuals who are on ART. Although clones of infected cells are present in untreated individuals, the confounding background of large numbers of proviruses in recently infected cells that have not clonally expanded makes it more difficult to use integration site analysis to identify the proviruses that are in clonally expanded cells.

Our results suggest that, in HIV-infected individuals on effective ART, expanded clones make up at least 40% of the infected cells [1], although, due to sampling limitations, this fraction is probably a considerable underestimate. In individuals on ART, a large majority of infected cells carry defective proviruses [6, 7]. Despite claims that viral expression would necessarily lead to cytopathic effects caused by the toxicity of viral proteins and/or immune clearance of the infected cells, which would preclude clonal expansion [8], it is now clear that some clonally expanded cells carry replication-competent proviruses and can release infectious virus into the blood [9]. There is accumulating indirect evidence that expanded clones that carry replication competent infectious proviruses are common [10–13]. Clones of infected cells appear to survive and expand, despite the toxicity of some of the viral proteins and immunological surveillance by the host, because, at any time, only a fraction of the cells that comprise the clones are producing viral RNA, and, by extension, viral proteins [9, 14]. As long as the overall proliferation of the cells in an infected clone equals or exceeds the clearance of the cells that express viral proteins, HIV infected clones can persist and can even expand over time.

Thus, clonal expansion of cells that carry replication-competent proviruses makes an important contribution to the generation and maintenance of the viral reservoir of infected cell, that persists despite long term ART. HIV infected cells that have clonally expanded can be detected by integration site analysis using DNA obtained from blood samples. However, only a small fraction (estimated to be about 2%) of the total T cell population is in the blood at any one time [15]. Lymph node biopsies have occasionally been obtained from HIV infected individuals, as have rare autopsy samples. There are, however, serious limitations on the samples that can be obtained from HIV infected people, and there are appropriate restrictions on experiments that can be done on humans. For those reasons, animal models of HIV infection have been developed. All the available animal models have limitations, which must be addressed to validate their relevance for asking specific research questions [16].

To evaluate the suitability of experimentally SIV-infected rhesus macaques as a model to investigate aspects of the clonal expansion of HIV-infected cells in humans, we first performed in vitro infections and compared the distributions of integration sites in HIV infected human PBMCs and SIV infected macaque PBMCs. Because this comparison is complicated by differences in the human and macaque genomes, and to ask whether there are differences in the overall distribution of the integration sites due to differences in the host cells (human vs. macaque), we also prepared a library of SIV integration sites from human PBMCs infected in vitro with SIV and compared the integration site distributions in these libraries to the distribution of HIV integration sites in human PBMCs (https://rid.ncifcrf.gov). Comparing the overall distributions of the integration sites, and the genes in which integration preferentially occurred, in the three libraries shows that the integration sites preferences of HIV and SIV are quite similar. These results confirm the conclusion, reached earlier based on a much smaller integration site dataset from a human cell line infected in culture with SIV [17].

Given the importance of clonal expansion of infected cells in the persistence of HIV in people on ART, we asked whether there was a similar clonal expansion of SIV infected cells in macaques. Samples were taken from 4 macaques that were infected for 4 weeks with SIVmac239, and then treated with a suppressive ART regimen for at least one year. The data show that the clonal expansion of SIV-infected cells in macaques is quite similar to the clonal expansion of HIV-infected cells in humans, that the timing of clonal expansion for infected cells is similar in HIV infected humans and SIV infected macaques, and that most of the largest SIV infected clones were found in more than one tissue in macaques.

## Results

### Integration site analysis of human and macaque cells infected in vitro with SIV

To assess the degree of similarity in the distributions of HIV and SIV integration sites in human PBMCs and SIV integration sites in macaque PBMCs, we stimulated human and macaque PBMCs, infected the cells with SIVmac239, and prepared integration site libraries from the infected cells using the methods of Maldarelli et al. [1](see also Materials and Methods). The library prepared from SIV infected human PBMC had 107,000 independent integration sites, and the library prepared from SIV infected macaque PBMC had 74,000 independent sites. The distribution of the SIV integration sites in these two libraries was compared to the distribution of 385,000 independent HIV integration sites in a library we previously prepared from infected human PBMC (https://rid.ncifcrf.gov). We did not attempt to prepare a library from HIV infected rhesus macaque PBMCs because species-specific viral restriction factors limit the efficiency of HIV infection of macaque PBMCs [18].

We began by comparing the overall SIV and HIV integration site preferences in human cells. This approach obviates the problem of trying to compare the integration sites in the genomes of different host species. A comparison of the fractions of integration sites present in genes, in association with CpG islands (+/- 1kB), and in association with transcription start sites (+/- 1kB) (Table 1), showed that the integration site preferences of SIV and HIV in the human genome were similar, confirming an earlier interpretation based on a much smaller number of SIV integration sites [17].

**Table 1:** SIV and HIV integration sites in relation to features in the human genome.

Integration sites and genomic landmarks	HIV-infected Human PBMC	SIV-infected Human PBMC	SIV-infected Monkey PBMC	MLV-infected Human CD34+	Random Sites
**Total Number of Integration Sites**	**(n = 385,205)**	**(n = 107,726)**	**(n = 52,712)**	**(n = 1,040,346)**	**(n = 259,648)**
**Percentage in RefSeq Genes**	**81.9**	**76.3**	**75.5**	**51.4**	**38.9**
**Percentage in CpG Islands (+/– 1 kb)**	**1.02**	**0.95**	**0.98**	**6.99**	**0.87**
**Percentage in TSS of RefSeq Genes (+/– 1 kb)**	**0.99**	**1.17**	**1.14**	**13.5**	**1.44**
**Median Gene Density in 1Mb region around the integration sites**	**15**	**12**	**12**	**10**	**5**

**Fig 1:**
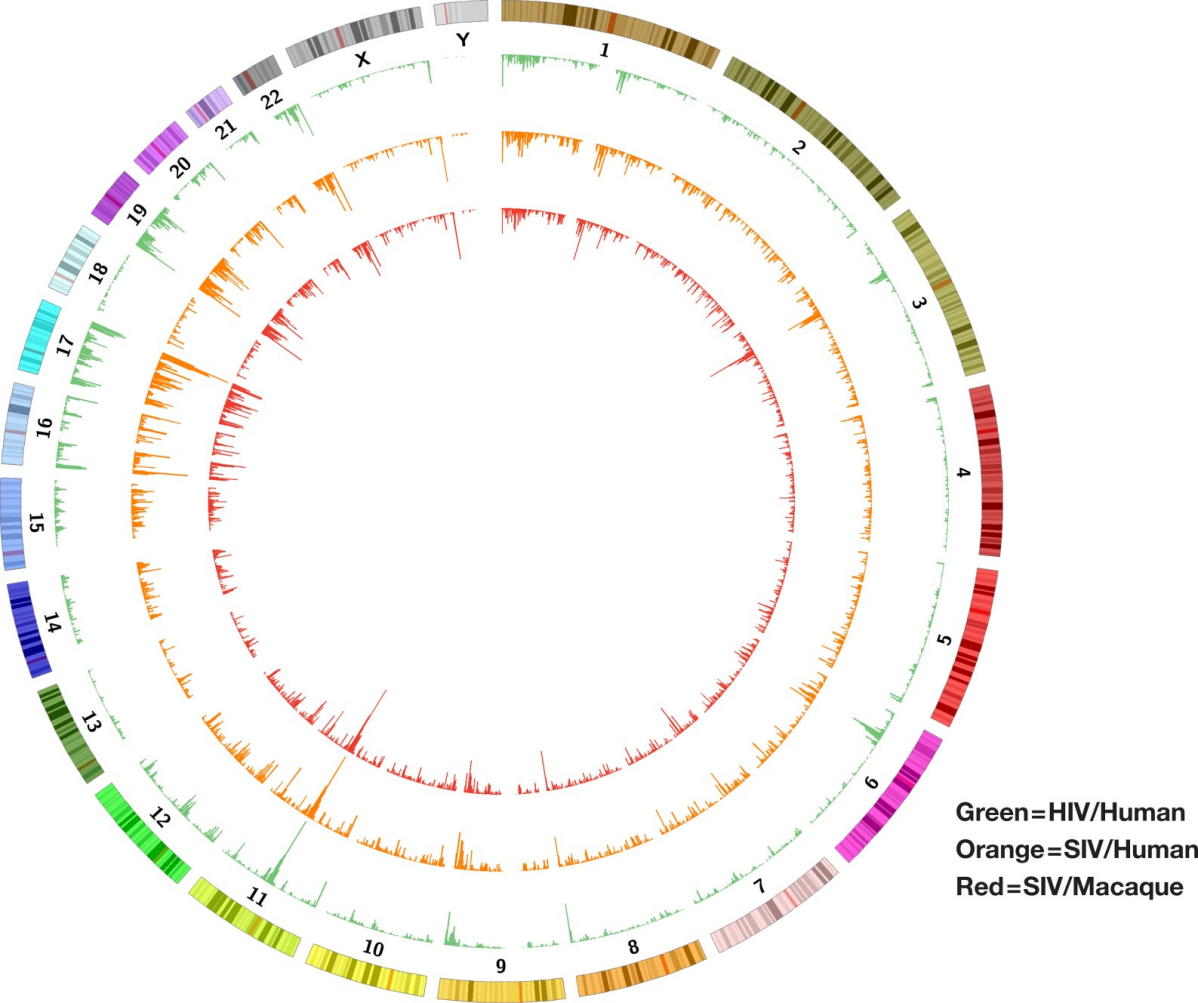
SIV integration sites in human and macaque PBMCs and HIV integration sites in human PBMCs. We prepared large integration site libraries from SIV infected human and macaque PBMCs, and we previously prepared a large library from HIV infected human PBMCs. To permit a direct comparison of the integration site data, the SIV/macaque integration sites were remapped onto the human genome (hg19, see text). Numbered chromosomes are indicated and the frequency of integrations in different loci is indicated the heights of the peaks, shown in green for HIV integrations in human PBMC, orange for SIV integrations in human PBMC and red for SIV integrations into macaque PBMC, mapped onto the corresponding loci in the human genome.

We also compared the integration site preferences of HIV and SIV in human PBMCs with the SIV integration site preferences in macaque PBMCs. We initially mapped the SIV integration sites onto the macaque genome (rheMac8). However, it is difficult to compare the relative distribution of integration sites in the two host genomes, particularly because the genes in the macaque genome are not as well annotated as the genes in the human genome. To address this problem, the SIV integration sites were mapped onto the human genome using the interval in the SIV genome +/- 50 bp on either side of the integration site. There is sufficient similarity in the sequences of the human and macaque genomes so that more than 70% of the >74,000 SIV/macaque integration sites (>52,000) could be mapped onto the human genome (see Materials and Methods). SIV had similar overall integration site preferences in human and macaque PBMCs (Fig 1 and Table 1).

**Fig 2:**
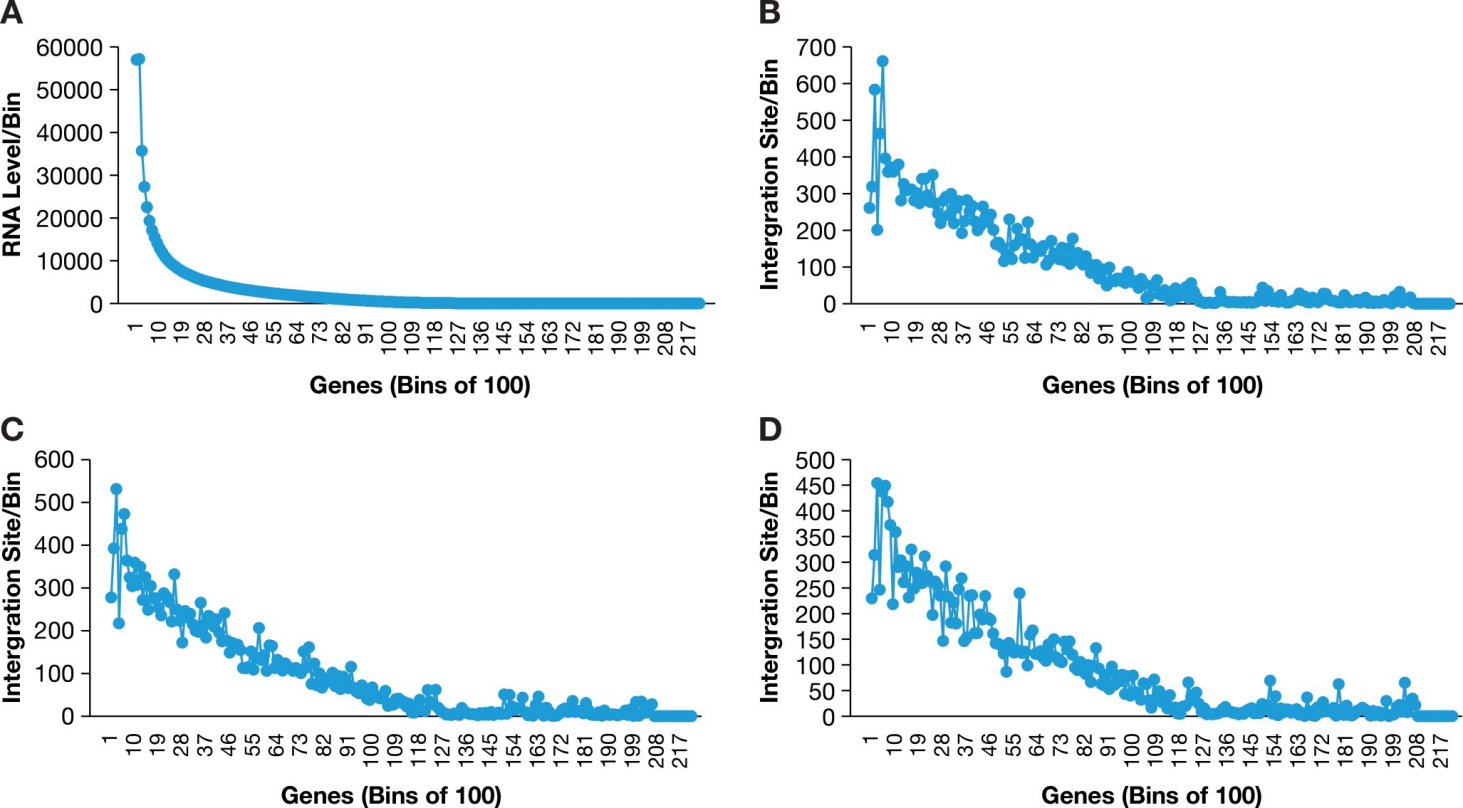
Both HIV and SIV preferentially integrate their DNA into highly expressed genes. Genes were placed in bins of 100, based on the levels of RNA in stimulated human PBMCs. Panel A shows the relative levels of RNA in each bin. Panel B shows the distribution of HIV integration sites in human PBMCs, placing the genes in the same bins as in panel A. Panel C shows the distribution of SIV integration sites in human PBMCs, using the same bins for the genes. Panel D shows the distribution of SIV integration sites into macaque PBMCs (the integration sites used in the analysis were mapped to the human genome).

It has been known for some time that HIV preferentially integrates into highly expressed genes [19]. To determine whether SIV has a similar preference, we used RNA-Seq to measure RNA levels in both human PBMCs and macaque PBMCs and showed that both viruses have a similar preference for integrating their DNA in highly expressed genes (Fig 2). Because our integration site datasets were large, we could also ask whether SIV and HIV preferentially integrated into the same subset of genes. We prepared lists of the 500 genes in which there were the greatest number of HIV and SIV integrations (Table 2). If we assume that there are approximately 25,000 genes in both species, and prepare two lists of 500 genes chosen at random, then the number of genes that would appear on both lists would be about 10, or 2% (after the first list of 500 genes has been chosen at random, there is a 1/50 chance that any gene chosen at random for a second list will be on the first list). However, when we did a comparison of the top 500 genes in the two libraries made from the infected human PBMCs, the number of genes in common in the two libraries was 300 (60%). As expected, the fraction of genes that were on both lists increased when lists of top 1000, 2000, or 5000 genes were compiled and compared (Table 2). To provide interpretive context for these observations, we made use of the fact that the 385,000 member HIV human PBMC library was created by infecting stimulated PBMCs from two different human donors (Donor 1, 225,000 integration sites; Donor 2, 160,000 integration sites). When the lists of the top 500 genes in the two HIV/human PBMC libraries were compared, there were 422 (84%) in common in the two libraries.

**Table 2:** Comparison of the genes that are the most frequent HIV and SIV integration targets.

Top 500 Gene Comparison				
Cell/Virus	Human/HIV	Human/SIV	Macaque/SIV	Random
**Human/HIV**	**500**	**300 (60%)**	**239 (48%)**	
**Human/SIV**		**500**	**323 (65%)**	
**Macaque/SIV**			**500**	
**Random**				**10 (2%)**
**Top 1000 Gene Comparison**				
**Cell/Virus**	**Human/HIV**	**Human/SIV**	**Macaque/SIV**	**Random**
**Human/HIV**	**1000**	**657 (66%)**	**567 (57%)**	
**Human/SIV**		**1000**	**693 (69%)**	
**Macaque/SIV**			**1000**	
**Random**				**40 (4%)**
**Top 2000 Gene Comparison**				
**Cell/Virus**	**Human/HIV**	**Human/SIV**	**Macaque/SIV**	**Random**
**Human/HIV**	**2000**	**1437 (72%)**	**1262 (63%)**	
**Human/SIV**		**2000**	**1395 (70%)**	
**Macaque/SIV**			**2000**	
**Random**				**160 (8%)**
**Top 5000 Gene Comparisons**				
**Cell/ Virus**	**Human/HIV**	**Human/SIV**	**Macaque/SIV**	**Random**
**Human/HIV**	**5000**	**4080 (82%)**	**3692 (74%)**	
**Human/SIV**		**5000**	**3809 (76%)**	
**Macaque/SIV**			**5000**	
**Random**				**1000 (20%)**
**Three-Way Overlaps**				
**Number of Top Genes**	**500**	**1000**	**2000**	**5000**
**Observed**	**203 (41%)**	**489 (49%)**	**1100 (55%)**	**3351 (67%)**
**All Random**	**0.2 (0.04%)**	**1.6 (0.2%)**	**12.8 (0.6%)**	**200 (4%)**
**Human Donor 1 Top Genes Compared to Human Donor 2 Top Genes**				
**Number of Genes**	**500**	**1000**	**2000**	**5000**
**Overlap**	**422 (84%)**	**881 (88%)**	**1778 (89%)**	**4592 (92%)**

**Fig 3:**
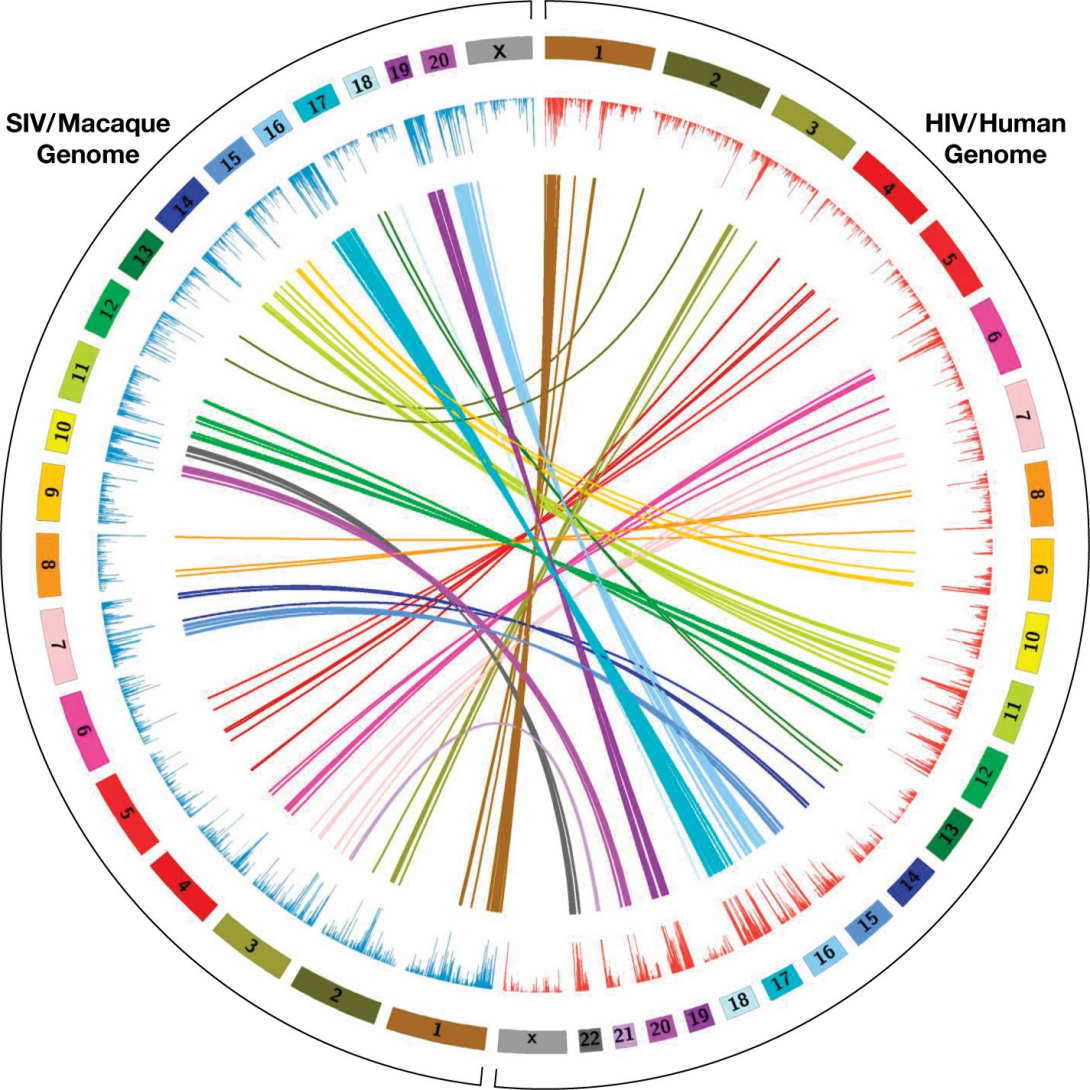
Preferred sites for SIV integration in macaque PBMCs and HIV integration in human PBMCs can be used to link syntenic regions of the human and macaque genomes. There are large syntenic blocks that can be used to connect homologous regions of the human genome (23 pairs of chromosomes) and the macaque genome (21 pairs of chromosomes). Regions of the macaque genome into which SIV preferentially integrates in PBMCs can be linked to the corresponding syntenic regions of the human genome by connecting the regions of the two genomes into which HIV and SIV preferentially integrate.

We also prepared a list of the top 500 genes into which SIV preferentially integrated in the macaque genome (mapped to the human genome) and compared it to the lists of the top 500 genes into which SIV and HIV preferentially integrated in human cells (Table 2). The number of top 500 genes in common between the SIV infected macaque and SIV infected human PBMC libraries was 323, or 65%. The overlap between the top 500 genes in the SIV infected macaque and HIV infected human PBMCs was 239, or 48%. As was true for the overlap in the top genes in the SIV infected human PBMC and HIV infected human PBMC libraries, the fraction of overlapping genes went up when the number of genes on the lists was increased to 1000, 2000, or 5000 genes (Table 2). We also determined the fraction of genes that were in the top 500, 1000, 2000, and 5000 in all three libraries. Again, the fraction that was present in all three libraries went up as the number of genes on the lists increased, from 41% for the top 500 genes to 67% when the top 5000 genes were compared. The lists of overlapping genes in the human and macaque libraries can also be used to connect the syntenic regions of the macaque and human genomes that comprise the gene-rich regions that are preferred targets for both HIV and SIV integration (Fig 3).

**Fig 4:**
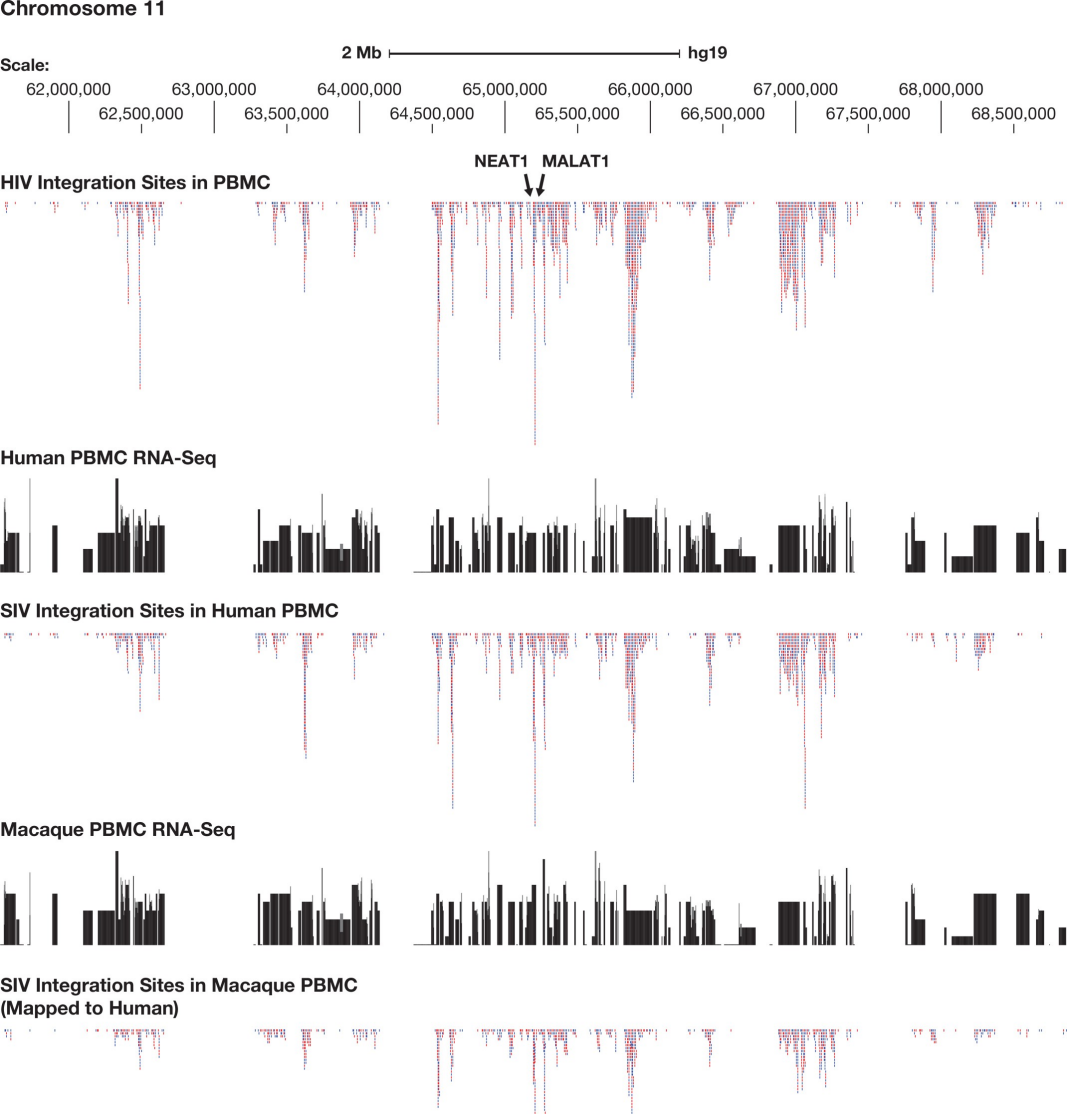
SIV and HIV preferentially integrate into regions that contain highly expressed genes. The figure shows a region of human chromosome 11 approximately 6 megabases in length that is centered around the long noncoding gene NEAT1. The top panel shows the distribution of 100,000 HIV integration sites in human PBMC, chosen at random from the 385,000 member library we prepared (reducing the number of HIV/human PBMC integration sites makes it easier to compare the data). The proviruses that are inserted in the same orientation as the numbering of the chromosome are shown in red, those in blue are in the opposite orientation. The second panel shows the human PBMC RNA-Seq data, mapped to the human genome, on a log scale. The third panel shows the distribution of SIV integration sites in human PBMC. The fourth panel shows RNA-Seq data from the macaque PMBCs, mapped to the human genome, and plotted on a log scale. The bottom panel shows the distribution of SIV integration sites in macaque PBMCs, mapped onto the human genome.

To ask whether the similarities in the overall distribution of the integration sites were also evident on a smaller scale, we performed selected comparisons of the integration site distributions in specific regions of the genome, for example in the region around the genes for the Neat1 and Malat1 long non-coding RNAs, which are on human chr 11, a region that is highly favored for both HIV and SIV integration. The corresponding/syntenic region of the macaque genome, on macaque chr 14, is also highly favored for SIV integration (see Figs 1, 3 and 4). These data confirm and extend the analysis that was done using large-scale comparisons and shows that the local distribution of integration sites is, like the overall distribution of integration sites, quite similar in the three libraries. The data also show that the genes that are expressed at high levels in macaque and human PBMCs are similar, and that both SIV and HIV preferentially integrate into the bodies of expressed genes. We also calculated the similarities in the overall distribution of the integration sites for the three libraries (Tables 3 and 4). All of the comparisons show that the distributions of SIV and HIV integration sites are quite similar. The substantial similarities among the in vitro integration sites in the three libraries make it possible to ask whether the limited differences seen in the distribution of the integration sites are due to differences in the target cell species (human vs. macaque), in the viruses (HIV vs. SIV), or both. Our analysis suggests that, although the overall patterns were quite similar, both the host and the virus make contributions to the modest differences in the integration site distributions (for example, in all of the comparisons of the top genes, the human/HIV and macaque/SIV libraries have the fewest genes in common) (Table 2).

**Table 3:** Correlation of the distribution of HIV and SIV integration sites (whole genome).

	HIV-huPBMC	SIV-huPBMC	SIV-rhePBMC	Random
**HIV-huPBMC**	**1**			
**SIV-huPBMC**	**0.801**	**1**		
**SIV-rhePBMC**	**0.687**	**0.813**	**1**	
**Random**	**0.0466**	**0.0541**	**0.0490**	**1**

Correlation by 100 kb window over the whole genome.

**Table 4:** Correlation of the distribution of HIV and SIV integration sites (by gene).

	HIV-huPBMC	SIV-huPBMC	SIV-rhePBMC	Random
**HIV-huPBMC**	**1**			
**SIV-huPBMC**	**0.831**	**1**		
**SIV-rhePBMC**	**0.700**	**0.810**	**1**	
**Random**	**0.138**	**0.167**	**0.177**	**1**

Correlation by gene.

### Analysis of in vivo integration sites

Having demonstrated that SIV and HIV have similar integration site preferences in cells infected in vitro, not only in terms of the distribution of integration sites near landmarks in the genome, but also in terms of which genes are the preferred targets, we determined the distribution of integration sites in PBMCs and tissue samples taken from SIV infected macaques. Samples were taken from 4 animals that were infected with SIVmac239 for 4 weeks, then placed on a suppressive ART regimen. Three of the animals received ART for approximately one year and were then euthanized; the fourth animal was maintained on ART for 90 weeks before being euthanized (see Materials and Methods). Blood was taken from all four animals at two and four weeks post-infection, prior to initiation of ART. Samples of blood, spleen, and axillary and mesenteric lymph nodes were taken from two of the animals after one year of treatment. A spleen sample and an axillary lymph node were taken from the third animal at one year and blood was obtained from the fourth animal at one year and at 90 weeks. The samples were analyzed for viral DNA loads (Table 5). Blood samples were taken and analyzed to determine the viral RNA load (Table 6).

**Table 5:** Levels of SIV DNA in samples taken from infected macaques.

		SIV DNA Copies per 10^6^ Cells			
Sample	Weeks of Infection,				
Type	Therapy Status	DCCN	DCHV	DCT3	DCJB
**PBMC**	**2 wks, pre-cART**	**154000**	**58300**	**43500**	**24300**
**PBMC**	**4 wks pre-cART**	**15100**	**14300**	**12300**	**17900**
**LN Mes**	**54 wks, on-cART**	**8450**	**2080**		
**LN Ax**	**54 wks, on-cART**	**5160**	**840**	**1690**	
**Spleen**	**54 wks, on cART**	**990**	**200**	**500**	
**PBMC**	**54 wks, on cART**	**2300**	**1100**	**440**	
**PBMC**	**90 wks on cART**				**No Sample**

**Table 6:** Levels of SIV RNA in samples taken from infected macaques.

		SIV RNA copies per	mL of plasma	
Weeks of Infection, Therapy Status	DCCN	DCHV	DCT3	DCJB
0.0 wks, pre-ART	*30*	*30*	*30*	*30*
**0.9 wks, pre-ART**	**2,200**	**3,700**	**2,700**	**9,800**
**1.3 wks, pre-ART**	**440,000**	**170,000**	**2,800,000**	**1,500,000**
**1.9 wks pre-ART**	**7,500,000**	**7,000,000**	**41,000,000**	**31,000,000**
**3.0 wks, on-ART**	**6,800,000**	**14,000,000**	**6,700,000**	**44,000,000**
**4.1 wks, on ART**	**430,000**	**3,500,000**	**2,400,000**	**23,000**

We obtained a total of approximately 8000 integration sites from the in vivo samples (Table 7). As expected, based on the number of infected cells present in each of the samples, we obtained the greatest number of integration sites from the blood samples taken after two weeks of infection (~5000), before the initiation of ART and at approximately the time of peak plasma viremia during primary infection (Table 6). Our ability to detect clones of infected cells is limited by sampling. Any of the samples we analyze contain only a small fraction of the infected cells present in the animals, and the methods we use can detect, at most, approximately 10% of the integration sites that are present in the samples [1]. We estimate that, in the animals on ART, clones must comprise >10^5^ cells to be detected using our standard assay (see Materials and Methods). Thus, integration site analysis underestimates the fraction of the infected cells that have clonally expanded.

**Table 7:** Number of total* integration sites in samples from four macaques.

	Integration Sites in PBMC	Integration Sites in PBMC	Integration Sites in PBMC	Integration Sites in Spleen	Integration Sites in AxLN	Integration Sites in MesLN	Integration Sites in PBMC
Animals	Pre-Therapy		On-Therapy				
	2 Weeks	4 weeks	12 Months				12 Months
**DCCN**	**1830**	**300**	**113**	**87**	**110**	**160**	**ND**
**DCHV**	**1239**	**84**	**48**	**189**	**116**	**236**	**ND**
**DCT3**	**1298**	**378**	**ND**	**218**	**126**	**ND**	**ND**
**DCJB**	**635**	**759**	**37**	**ND**	**ND**	**ND**	**105**
**Totals**	**5002**	**1521**	**198**	**494**	**352**	**396**	**105**

* the total count of integration sites includes counting the same site more than once, if it was recovered more than once from a clone of expanded cells. Thus, these counts are higher than the counts of unique sites in S1 Table.

A total of 107 clones were identified in samples obtained from the animals (**S1 Table**). 101 of those clones were found in the on-ART samples. Almost 20% of the total integration sites in the on-ART samples (19.4%, 299 of 1545) were shown to be in clonally expanded cells. Because after ART has been initiated, few if any additional cells are newly infected, the cells that gave rise to the expanded clones found in the on-ART sample were likely to have been infected before therapy was initiated. Cells from 5 of the 7 largest clones (those in which the integration site was isolated 6 or more times) were present in more than one of the tissues (Table 8). However, there was one clone for which we isolated the same integration site 10 times; all were in a sample taken from a single lymph node.

**Table 8:** Distribution of the largest clones of integration sites in different tissues after one year of ART.

Animal	Integration Site	Gene	Blood	AxLN	MesLN	Spleen
**DCCN**	**Chr17: 27,611,290**	**RB1**	**+**	**+**		**+**
**DCCN**	**Chr19: 806,326**	**CNN2**			**+**	
**DCHV**	**Chr:10: 61,161,726**	**UFD1L**			**+**	
**DCHV**	**Chr15: 81,246,544**	**RFX3**	**+**	**+**		**+**
**DCT3**	**Chr8: 144,060,762**	**ZNF34**		**+**	**ND**	**+**
**DCT3**	**Chr11: 1,422,670**	**ERC1**		**+**	**ND**	**+**
**DCT3**	**Chr12: 85,428,795**	**CD28**		**+**	**ND**	**+**

To better understand the origin of the clones of SIV-infected cells that were identified in animals on effective ART for one year, we analyzed the integration sites in blood samples that were taken from the four animals two weeks and four weeks after the initial infection, prior to the initiation of ART (Table 7). Note that there are differences in the interpretation of pre-ART and on-ART integration site data. It is possible, in some cases, that integration sites were obtained from single cells that had recently replicated their DNA, but not yet divided. When, in an on-therapy sample, we obtain the same end of a provirus two or more times, we identify that site as being in a clone of expanded cells. If there are infected cells that have replicated their DNA, but not yet divided, in samples taken after a year or more on-ART, it is quite likely that the cell is dividing, and is part of a clone. However, in untreated SIV infected macaques, as in untreated HIV-infected humans, most newly infected cells die shortly after infected individuals are put on ART [16]. This large background of newly infected cells makes it much more difficult to identify clones of infected cells in pre-ART samples. In addition, any clones that are present in a pre-ART sample taken shortly after the initial infection might not have had time to expand to include a sufficiently large number of cells to be detected as clones. Moreover, in samples taken from untreated macaques (or people), some of the recently infected cells could be in the S or G2 phases of the cell cycle. In experiments done with cells infected in culture, expression of either HIV-1 or SIV Vpr arrested cell division in G2 [20–22]. It is not clear whether there is an equivalent G2 arrest of infected cells in vivo, and, if there is, how long the arrested cells survive, but it is unlikely that they will divide and grow into clones. Nevertheless, such cells would contain two copies of the same provirus, which could allow us to isolate the same end of the provirus with two host DNA breakpoints in the integration site assay. For that reason, it is unclear, when we have obtained the same end of a provirus with two different breakpoints from a pre-therapy sample, whether that integration site was obtained from two infected cells or a single cell which carried two copies of the same provirus. Because the pre-therapy and on-therapy integration site data are somewhat different, and the ways in which clones are identified are different in the two datasets, the data are not strictly comparable, and are reported separately in Tables 9 and 10 (see Discussion, and Materials and Methods).

**Table 9:** Pretherapy PBMC viral DNA and integration sites.

Monkey	SIV DNA*		Unique Integration Sites		Sites with >1 Breakpoint		Confirmed Clones		Fraction of the Integration sites in Confirmed Clones	
	2 weeks	4 weeks	2 weeks	4 weeks	2 weeks	4 weeks	2 weeks	4 weeks	2 weeks	4 weeks
**DCCN**	**154,000**	**15,100**	**1830**	**300**	**5**	**2**	**0**	**1**	**< .0005**	**0.0033**
**DCHV**	**58,300**	**14,300**	**1239**	**84**	**8**	**0**	**0**	**0**	**< .0004**	**< .02**
**DCT3**	**43,500**	**12,300**	**1298**	**378**	**4**	**10**	**1**	**2**	**0.00077**	**0.0053**
**DCJB**	**24,300**	**17,900**	**635**	**759**	**3**	**10**	**0**	**3**	**< .002**	**0.0039**
**Total or mean**	**93,300**	**14,900**	**5002**	**1521**	**20**	**22**	**1**	**6**	**0.0002**	**0.004**

*Lymph-Average of data from the axillary nodes, mesenteric nodes, and spleen for DCCN and DCHV and the axillary node and spleen for DCT3

**Table 10:** On-therapy viral DNA and integration sites.

Monkey	SIV DNA*		Unique Integration Sites		Clones		Fraction of the Integration Sites in Clones	
	PBMC	Lymphatic	PBMC	Lymphatic	PBMC	Lymphatic	PBMC	Lymphatic
**DCCN**	**2300**	**4870**	**100**	**309**	**13**	**27**	**0.13**	**0.087**
**DCHV**	**1100**	**1040**	**45**	**486**	**4**	**30**	**0.089**	**0.061**
**DCT3**	**440**	**730**	**120**	**174**	**ND**	**27**	**ND**	**0.16**
**DCJB**	**ND**	**ND**	**628**	**872**	**20**	**ND**	**0.031**	**ND**
**Total or mean**			**893**	**1841**	**37**	**84**	**0.048**	**0.087**

*Lymph-Average of data from the axillary nodes, mesenteric nodes, and spleen for DCCN and DCHV and the axillary node and spleen for DCT3

We identified one clone of SIV infected cells in a sample taken after the animal had been infected for only 2 weeks. This integration site was identified as being in a clone based on finding the same integration site in two different types of samples, obtained at different times. In this case, the integration site identified in a PBMC sample at 2 weeks post infection, prior to ART, was also found in a tissue sample taken one year later while on ART (**S1 Table**). We found 6 clones in samples taken at 4 weeks, although there were many fewer overall integration sites in the 4-week samples (about 1500 vs. about 5000; plasma viremia was declining by 4 weeks, compared to 2 weeks, Table 6). In addition to the one site that could be verified as belonging to a clone, samples taken from all 4 macaques at 2 weeks and samples taken from 3 out of 4 macaques at 4 weeks contained sites with exactly 2 breakpoints. The frequency of such sites increased from about 0.4% at week 2 to 1.4% at week 4 (Table 9), in contrast to the 7-fold overall decline in SIV DNA and greater than 3-fold decline in integration sites recovered. At this time, we cannot determine which of the two possibilities discussed above explains how two breakpoints were obtained for these integration sites, and although we cannot rule out their possible origin from cells that had replicated their provirus containing DNA but not yet divided, the increase in their frequency between the two time points is consistent with growth of some infected cells into clones large enough for us to detect by two weeks after the initial infection.

Because preferential growth or survival of a fraction of HIV infected cells in humans has been linked to the integration of HIV proviruses in specific introns of the *BACH2*, *MKL2*, *and STAT5B* genes [1, 2, 23], we looked for SIV proviruses integrated in these genes. We found a total of 6 proviruses in *BACH2*, 3 in *MKL2*, and 9 in *STAT5B*. Table 11 describes the integration sites in these three genes in the four infected macaques, and compares the macaque data to the integration sites found in these three genes in an HIV-1-infected patient on suppressive ART [1]. Most of the in vivo SIV integration sites (ca. 6500 out of about 8000) were obtained from the 2- and 4-week samples, which were taken from untreated animals. Based on the very small number of clones we detected in the pre-ART samples, we did not expect to find any clones whose replication was driven by proviruses integrated into *BACH2*, *MKL2* or *STAT5B* in the 2- and 4-week pre-ART samples. Of the 9 SIV integration sites in *BACH2* and *MKL2* in the macaque samples, only 3 were in the on-ART samples. Only one site (in *BACH2*, on-ART), was in the same region (*BACH2* intron 5) and the same orientation (same as the gene) as the integration sites that were selected in Patient 1. None of the 9 integration sites was in a detectable clone of expanded cells.

**Table 11:** SIV integration sites in genes where integration has been linked to clonal expansion in an HIV-infected patient.

Gene Pre^b^ or On^c^ ART	SIV Integration Sites in 4 Macaques			HIV Integration Sites in Patient 1 ^a^		
	Total Sites	Intron, Provirus Orientation ^d^	Number of Sites in Each Intron	Total Sites	Intron, Provirus Orientation ^d^	Number of Sites in Each Intron
**BACH2 Pre**	**5**	**1-,3+,3+,4-, 5-**	**1,2,1,1**			
**BACH On**	**1**	**5+**	**1**	**15**	**4,5, All +**	**2,13**
**MKL2 Pre**	**1**	**10-**	**1**			
**MKL2 On**	**2**	**2-,2-**	**2**	**15**	**4,6, All +**	**4,11**
**STAT5B Pre**	**5**	**1-,1-,1+,1+,3-**	**4,1**			
**STAT5B On**	**4**	**1+,1+,1+,1-**	**4**	**3**	**1+, 1-,1-**	**3**

a From Maldarelli et al (2014) (1124 total)

b PBMC at 2 or 4 weeks post infection (6523 total)

c PBMC or lymph nodes at 12 or 20 months post infection (1545 total)

d + is in the same orientation as the gene,—is opposite to the gene

HIV integration in the first intron of the *STAT5B* gene, in the same orientation as the gene, has also been linked to clonal expansion of the infected cells [23]. However, in contrast to *BACH2* and *MKL2*, which are not preferential targets for HIV integration, *STAT5B* is a preferred target for HIV integration, which complicates the analysis. We found 5 SIV proviruses integrated in *STAT5B* in samples taken from infected animals before ART was initiated, and 4 additional proviruses in samples taken after a year of ART treatment. In the on-ART samples we analyzed, there were too few integration sites in *STAT5B* to determine if there was a selection for integration sites in *STAT5B* on-ART. We did find one expanded clone in which an SIV provirus was integrated in the first intron of the *STAT5B* gene, in the same orientation as the gene, which is the region of the gene and the orientation of the provirus that is associated with clonal growth of HIV infected T cells in humans.

We did a similar comparison of the fractions of integration sites in the pre-ART PBMC dataset and the on-ART dataset for all of the genes to look for evidence of selection for proviruses integrated into any other genes in the macaques on ART. Because the current on-ART dataset of SIV integration sites is relatively small, there were only a few integration sites for most of the genes, and we were not able to find evidence of selection for integration sites in any gene that was statistically significant. The largest SIV infected clone (judged by the number of times the integration site was recovered) had an SIV provirus inserted in the *RB1* (retinoblastoma) gene in the opposite orientation relative to the gene. *RB1* is a frequent target for SIV integration *ex vivo*. 43 SIV proviruses were found in the *RB1* gene in the rhesus PBMC infected in vitro. We identified only one clone in which there was an SIV provirus integrated in *RB1* in any of the on-ART samples and there were 3 proviruses found in *RB1* in the pre-therapy samples. Thus, the data we have do not provide any evidence that SIV integration into the *RB1* gene is connected to the clonal expansion and persistence of the infected cells.

**Fig 5:**
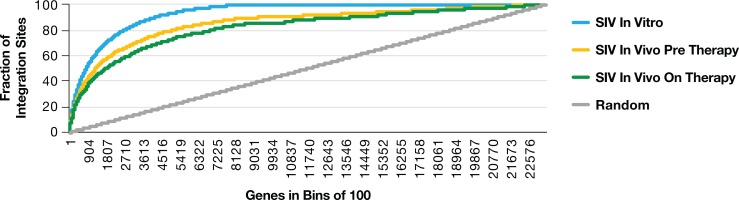
The overall distribution of SIV integration sites in macaque PBMC infected in vitro is similar to the distribution of integration sites in samples taken from infected macaques pre-ART (both 2-week and 4-week samples) and on-ART. For the in vivo pre-ART and on-ART analysis, the data from all the infected macaques was combined. Genes were placed in bins of 100 based on the frequency of integration sites in the genes in SIV infected macaque PBMC. Each point on the Y axis represents the cumulative fraction of integration sites in all of the bins to the left of that point of the X axis. The in vitro integration site data are shown in blue, the in vivo pre-ART data are shown in yellow, the in vivo on-ART data are shown in green, and a computer-generated random control data are shown in gray.

**Fig 6:**
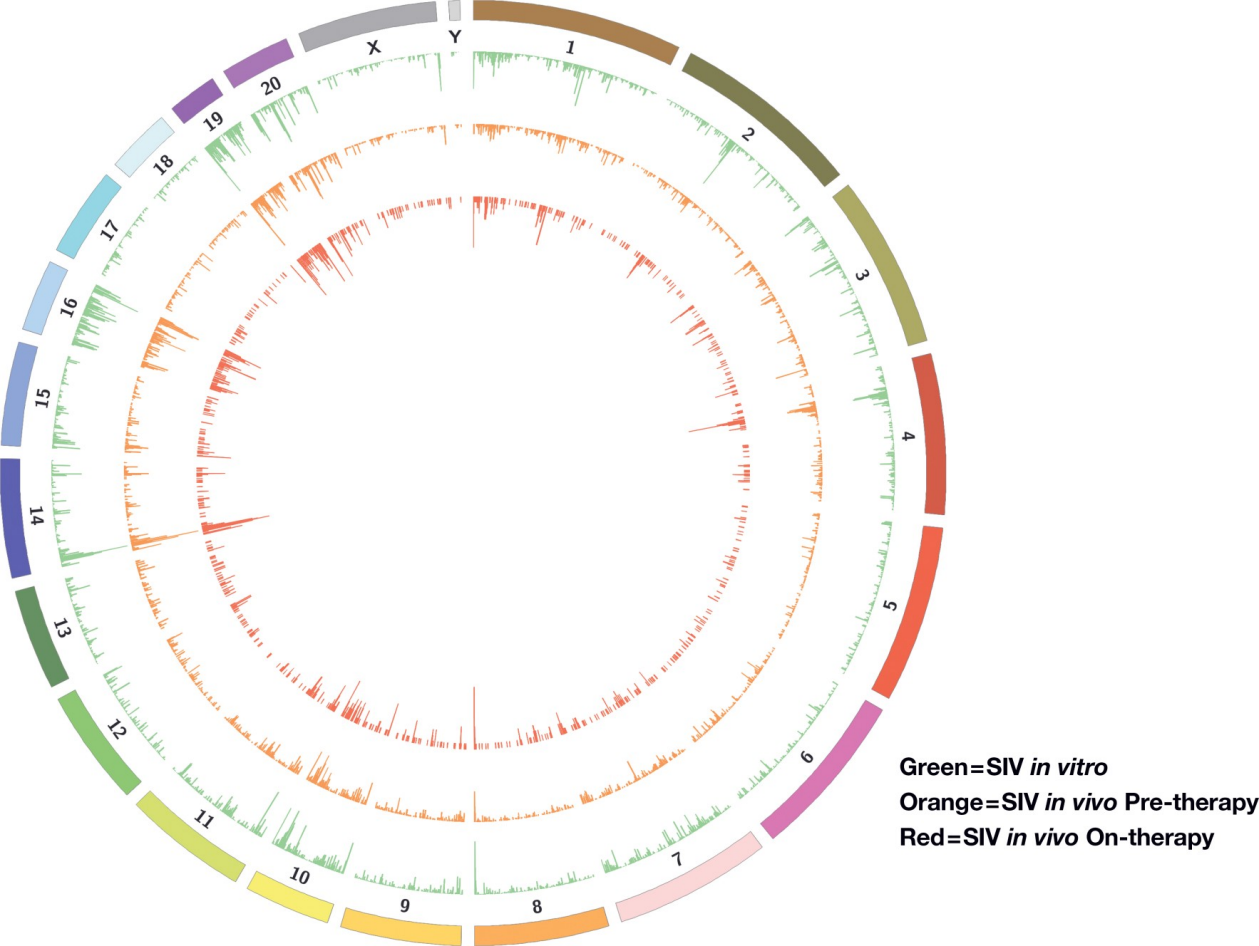
The overall distribution of SIV integration sites in macaque PBMC infected in vitro, and mapped to the macaque genome, is similar to the distribution of the SIV integration sites in samples taken from all of the SIV infected macaques both pre-ART (both 2-week and 4-week data) and on-ART. The integration site data from the macaque PBMC infected in vitro are shown in green. The combined pretherapy integration site data are shown in orange, and the combined on-ART integration site data are shown in red.

We also compared the overall distribution of integration sites in the in vitro SIV infected PBMC, the (combined) pretherapy samples, and the (combined) on-ART samples (Figs 5 and 6). These analyses show that, as expected, the overall distribution of integration sites was quite similar in vivo and in vitro. The data also show that the initial distribution of integration sites changed only modestly during a year on ART. This result supports the conclusion that there is, at most, only a limited selection for proviruses that are integrated into specific genes.

We looked in samples taken from SIV infected macaques for evidence of a selection for/against proviruses in either orientation relative to the host gene in which they are integrated. In human cells infected with HIV in culture, there is no initial bias in the orientation of the proviruses relative to the gene. However, in samples taken from HIV patients on therapy, there were significantly fewer proviruses integrated in the same orientation as the gene, relative to those in the opposite orientation [1]. We analyzed the orientations of SIV proviruses relative to the orientation of the host genes in macaque PBMCs infected in vitro and in samples from infected animals taken both pre-therapy and on-therapy. As was seen in the HIV/human data, there was no bias in the orientation of SIV proviruses in recently infected cells in tissue culture. There was a modest bias against the proviruses in the same orientation as the host gene in the pre-therapy samples, and there were significantly fewer SIV proviruses in the same orientation as the gene in the on-therapy samples (Table 12). Proviruses in the same orientation as the gene are more likely to interfere with normal gene expression than are proviruses integrated in the opposite orientation, which would explain the selection against proviruses that are integrated in the same orientation as the gene (see Discussion).

**Table 12:** Orientation of SIV integration sites relative to the surrounding gene.

Infected Cells	Same as Gene	Opposite to Gene	Ratio Same/ Opposite	P-value*
**PBMC (in vitro)**	**37024**	**36946**	**1.00**	
**Pre-therapy**	**3178**	**3345**	**0.95**	**0.040**
**On-therapy**	**705**	**840**	**0.84**	**0.00062**

*Fisher Exact Test

## Discussion

Animal models of human disease allow experimental questions to be addressed that cannot be readily studied in a clinical setting. Animal models of HIV infection in humans, including SIV-infected macaques, are being used to study the origin and behavior of the reservoir that persists as DNA copies of the viral genome, integrated in the genomes of infected T cells [16]. This viral DNA persists for the life of infected individuals despite ART that effectively suppresses viral replication. While most are defective, some of these proviruses are intact and represent part of the reservoir of infected cells that gives rise to recrudescent infection when ART is interrupted. We and others have shown that there is extensive clonal amplification of HIV infected cells in those who are infected with the virus [1, 2]. Despite a claim to the contrary [8], we also showed that expanded clones can carry an infectious provirus and release detectable levels of virus into the blood [9]. Thus, clonal expansion of cells that carry infectious proviruses makes an important contribution to the generation and persistence of the viral reservoir that has made it impossible to cure HIV infections with drugs that block HIV replication. These observations have important implications for strategies intended to reduce or eliminate the reservoir.

In the present study, we used integration site analysis to ask whether a similar clonal expansion of SIV infected cells occurs in macaques, and whether SIV-infected macaques on suppressive ART represent a relevant and useful model to study the clonal expansion of HIV infected cells. Before embarking on a comparison of in vivo integration site data and its implications for the clonal expansion of infected cells, we confirmed and extended an earlier report, based on analysis of 148 integration sites, that SIV and HIV have similar patterns of integration in cells infected in vitro [17]. We generated large integration site libraries from human and macaque PBMCs that were infected in vitro with SIV and compared the distribution of the SIV integration sites to the distribution of integration sites in a similar library prepared from human PBMCs infected in vitro with HIV (https://rid.ncifcrf.gov). The advantage of comparing the data from the in vitro libraries is two-fold. First, because samples obtained from SIV infected macaques, or HIV infected humans, contain, at most, a few infected cells in a thousand, much larger libraries can be prepared from cells infected in vitro than from in vivo samples. Second, because the timing of the infection can be controlled in vitro, the experiments can be done in a way that precludes the possibility of extensive selection for or against infected cells with integration sites in or near specific genes, and/or in specific orientations, which could perturb the distribution of the integration sites. A comparison of the integration site distributions in the SIV infected human and macaque PBMCs with HIV integration sites in human PBMCs showed that SIV and HIV have similar integration site preferences, that the overall distribution of HIV and SIV integration sites is quite similar, and that the genes that are the preferred targets in humans and macaques are also very similar. This suggests that the HIV and SIV pre-integration complexes (PICs) interact with the same host factors as the PICs transit the cytoplasm, cross the nuclear membrane, and move through the nucleus to associate with the chromatin that contains the preferred integration sites [24–27]

### Cells infected with SIV in the first few weeks can clonally expand

The HIV and SIV viral RNA and DNA load, typically measured in plasma and cells obtained from the peripheral blood, usually reaches a peak within the first 2–3 weeks after the initial infection (Tables 5 and 6). The viral loads decline as target cells are depleted and the host’s immune system begins to recognize infected cells that express viral proteins [28, 29]. If infected humans (and macaques) are put on ART, there is an additional decline in the viral DNA load because infected cells continue to die but are not replaced by new infections [28]. Both immune surveillance and the toxicity of viral proteins favor the accumulation of infected cells (and clones) that either carry extensively defective proviruses or intact proviruses that are either not expressed or are expressed at a very low level. Nevertheless, HIV-infected humans can have large clones that carry infectious proviruses and these clones can release detectable levels of infectious virus into the blood [9]. On ART, the viral DNA load eventually stabilizes although it may continue to decline very slowly [30, 31], presumably because cells infected with defective and/or latent proviruses are not subject to immune clearance or viral cytopathology, and because there is sufficient growth and expansion of some of the clones of infected cells to balance (or nearly balance) any additional loss of infected cells.

In the current study, we recovered fewer integration sites from the pre-ART SIV infected macaque blood samples taken 4 weeks after the animals were infected than from pre-ART samples taken after 2 weeks of infection. However, because there was much less viral DNA present at 4 weeks, relative to 2 weeks, the fraction of the sites we recovered was greater. We obtained even fewer sites (but a still larger) fraction of the sites that were present) from the samples taken after the animals were receiving ART for one year relative to the 4-week samples. These data reflect the increasing proportion of long-lived cells that contain proviral DNA in the 4 week and on-ART samples. (Tables 9 and 10). Although we obtained a considerably higher total number of integration sites from the pre-therapy samples, the majority of the pre-therapy integration sites were likely to have been derived from recently infected cells. Using stringent criteria to identify clones in the pre-ART samples, we identified only one confirmed clone in the 2-week samples (0.02% of total sites) and only 6 confirmed clones in the 4-week samples (0.4%). The clone identified in the 2-week sample was identified because we found a provirus with the same integration site in a sample isolated at one year on-ART from the spleen of the same animal, showing that it had grown into at least a small clone by 2 weeks and that clones that arise early can persist. These data suggest that the earliest infected clones can grow large enough to be readily detected between 2 and 4 weeks after infection, in good agreement with our data for the time it takes HIV infected clones to grow to a detectable size in humans. We compared the time at which clones of SIV infected could first be detected in samples taken from untreated macaques with data from humans that were recently infected with HIV for approximately the same length of time, based on Fiebig staging [32, 33]. We also compared the fraction of SIV infected cells that we could show had clonally expanded in the 4-week sample to the fraction of clonally expanded HIV-1 infected cells we detected in untreated humans who had been infected for a similar length of time. Both the times at which the clones grew to a detectable size, and fraction of integration sites that were in clones, were similar.

In addition to the sites we found in the untreated macaques that could be confirmed as belonging to clonally expanded cells, there were about 20 integration sites for which we obtained 2 breakpoints at both 2- and 4-weeks post infection (0.4% and 1.4%, respectively). As noted in the Results, We were cautious in interpreting the data in part because the fraction of infected cells that carry two proviruses integrated at the same site may be enhanced by Vpr-mediated G2 arrest [20–22]. The >3-fold increase in frequency with which we detected integration sites with two breakpoints increased between 2 and 4 weeks, taken together with the demonstrated presence of clonally expanded cells at 4 weeks, suggests that we may have underestimated the number of clones in the pretherapy samples. We saw a similar increase in detectable clones in samples taken from humans sampled at similar times after infection [34].

In both SIV infected rhesus macaques and in HIV infected humans, the reservoir is known to be established within a few days of the initial infection [28, 35, 36]. Thus, if clonal expansion of infected cells carrying replication competent proviruses is an important part of the establishment and persistence of this reservoir, and ART blocks viral replication, it is important to show that the clones that comprise the reservoir are derived from cells that are infected before ART was initiated. However, even if all of the cells that gave rise to the expanded clones identified in samples taken after initiation of ART were infected early, many of those clones might not have grown large enough to be detected as early as 4 weeks after infection. We estimate, if we recover approximately 1000 integration sites from an on-ART sample, that clones must have expanded to >10^5^ cells for us to have a good chance to detect them in our standard assay. If we get fewer integration sites, the clones must be larger for them to be readily detected; with more integration sites we can expect to detect smaller clones.

The doubling time of infected activated CD4+ T cells in vivo is not known. If we assume it to be 1 day, it would take more than 2 weeks a for single infected cell to grow into a clone of 10^5^ cells. However, a shorter doubling time has been estimated for activated CD4+ T cells in mice that were responding to a viral antigen [37], a situation that resembles early HIV and SIV infection. If we assume a doubling time of one day, given the low levels of virus detected in the blood of infected macaques about a week after infection, and the rapid rise in the viral load in the blood in the second week of infection (Table 6), we would not have expected that there would be clones large enough for us to detect in the samples taken two weeks after the animals were infected, particularly given the large background of newly infected cells. If, on the other hand, the doubling time of human and macaque CD4+ T cells is closer to 11 hours (the time estimated from the mouse data), then it would only take about 1 week for a single infected cell to become a clone large enough for us to detect. The data we present make it clear that there are infected cells that have grown into large clones by 4 weeks of infection. The data also suggest that there may be large clones that arise earlier; however, additional data will be needed to properly test that possibility.

100 additional clones were found in samples taken after one year of ART. Although some controversy remains, it is well-established that effective ART greatly reduces, or more likely eliminates, new rounds of productive HIV infection in humans and SIV infection in macaques [3–5]. Thus, the integrated proviruses we recovered from the samples taken from macaques after one year of ART were likely to be in cells that were infected before ART was initiated, or in their clonal descendants. Because the number of integration sites we recover also affects the fraction we can assign to clones, it is likely that the fraction of integration sites shown to be in clones in the SIV/macaque data (about 4–10%; Table 10) is lower than the fraction in the HIV/human on-therapy sample from patient 1 of Maldarelli et al. [1] (approximately 40%) because the number of integration sites we recovered was lower for the SIV/macaque on-ART samples, although the much longer time on ART (ca. 11 years) for patient 1 may have contributed to the difference.

What causes HIV infected cells to clonally expand and persist? In humans, there is compelling evidence that integration of an HIV provirus in specific regions (1 or 2 specific introns) of the *MKL2*, *BACH2*, *and STAT5B* genes, and in the same orientation as the gene, is associated with clonal expansion, although the exact underlying mechanism(s) remains to be elucidated [1, 2]. The available data suggest that such proviruses affect the expression of these genes, and possibly the structure of their protein products, in a way that confers some proliferative or survival advantage to the cells.

Proviruses integrated in *MKL2*, *BACH2*, and *STAT5B* genes represent only a small fraction (<3%) of the total proviruses in patients on long term ART, and there are other mechanisms, such as antigenic stimulation and homeostatic cytokine signaling that can contribute to the clonal proliferation and long-term survival of HIV (and SIV) infected CD4+ T cells. The relative contribution of these two mechanisms to the overall clonal expansion can, in theory, be answered by comparing the distribution of integrated proviruses in a sample taken from an acutely infected individual (before there is a significant opportunity for selection) with one taken after long-term therapy. However, as noted earlier, we cannot obtain a sufficient number of integration sites from in vivo samples to accurately determine the initial distribution of the integration sites in acute infection relative to the ~25,000 genes in the genome. Comparison of a large library (385,000 integration sites) made from PBMCs from normal human donors that were infected in vitro with HIV with the distribution of a more limited number of integration sites obtained from acutely infected people showed that the overall distributions of the integration sites are quite similar [34]. This result supports the use of in vitro libraries as surrogates for the initial distribution of integration sites in vivo. We also found that the initial distribution of HIV integration sites was largely preserved even after years of successful ART, despite the selection for HIV proviruses integrated in *MKL2*, *BACH2*, or *STAT5B*. Based on what is known about the biology of T cells, it is likely that the majority of the infected T cells clonally expand in response to antigenic and/or homeostatic/cytokine stimulation, rather than due to the integration of proviruses in particular genes.

We did a similar analysis of the data from SIV infected macaques to look for evidence for selection of cells with proviruses integrated into the *BACH2*, *MKL2*, and *STAT5B* genes in SIV infected rhesus macaques. In the on-ART data set, we found only a single provirus that was integrated in the region of *BACH2* and in the same orientation relative to the gene as the HIV proviruses that were selected in infected humans. There were 3 proviruses in the region of STAT5B in the orientation that has been associated with the clonal expansion of HIV infected T cells in humans. One of these proviruses was shown to be in a clone of expanded cells. While tantalizing, this single SIV provirus is not sufficient to reach any conclusion. As was the case with *BACH2*, the data for *STAT5B* and *MKL2* were not sufficient to show a relationship between SIV integration into these genes and clonal expansion of cells in macaques. Nor did we find evidence linking SIV integration in any other gene with clonal expansion and/or persistence of the infected cells in macaques. However, thus far we have characterized relatively few integration sites from SIV infected macaques on ART. It is possible that the clones that expanded due to HIV integration only grow large enough for us to detect after several years of therapy [see, for example, Table 1 in Maldarelli et al. [1]]. Evidence connecting SIV integration to clonal expansion and persistence of infected cells in macaques may yet emerge as we extend our analyses to include more sites and longer times on ART.

However, if, as the data for HIV infected cells in humans strongly suggest, the primary mechanism that causes the clonal expansion in SIV infected cells in macaques is not integration into specific genes, but instead involves antigen driven or homeostatic proliferation of infected cells, then the overall distribution of SIV integration sites should be minimally affected by long-term ART. We first showed that the distribution of integration sites in the in vitro library made by infecting macaque PBMCs with SIV and the integration sites in the samples taken from the macaques before ART was initiated were similar. We then asked if the distribution of the SIV integration sites seen in macaque PBMC infected in vitro was also seen in the samples taken after one year of ART. The results were quite similar to those obtained from a similar analysis comparing the distribution of HIV integration sites in PBMC infected in vitro to the integration sites in samples taken from humans on ART. In both systems, within the limits of the sampling and analysis, the initial distribution of integration sites was well preserved on ART. The SIV/macaque results support the conclusion that much of the clonal expansion of infected cells is not driven by integration into specific genes but is rather due to homeostatic/cytokine and/or antigen driven proliferation. Thus, the unresolved question of whether SIV integration into specific regions of *BACH2*, *MKL2*, *STAT5B*, or some other gene does (or does not) drive the expansion and/or persistence of a small fraction of the SIV infected clones in macaques is an interesting but relatively minor point in determining the relevance and utility of the SIV/macaque model.

Although we did not find any strong evidence of selection for proviruses in specific host genes, we did see evidence of selection favoring proviruses integrated in the opposite orientation relative to the targeted host gene (Table 12), compared to proviruses integrated in the same orientation, a bias we also saw in HIV infected cells in patients on ART [1]. As noted earlier, it is likely that the selection against the proviruses integrated in the same orientation relative to the host gene are the result of sequences in the proviruses, such as splice sites and polyadenylation sites, interfering with the expression of the host gene. Because many of the proviruses in HIV infected humans and SIV infected macaques are defective, and their defects vary considerably, the specific consequences of inserting a particular defective provirus in any given intron of a host gene are difficult to predict. The breadth of selection against proviruses in the same orientation as the gene must mean that the presence of at least some types of defective proviruses in a number of host genes negatively affect growth or survival of the infected cell, despite the provirus being present in only one of the two copies of the host gene. Both positive and negative selections involving proviruses in a specific orientation relative to the host gene are well documented for other retroviruses. Examples involve tumor induction and the long-term survival of endogenous proviruses [38–40].

Overall, the results obtained in our studies of the clonal expansion of SIV infected cells in rhesus macaques suggest that the clonal expansion of HIV infected cells in humans can be effectively modeled in this nonhuman primate system. For example, in both untreated HIV infected humans and untreated SIV infected macaques, the vast majority of the infected cells are newly infected, and have not clonally expanded, which makes it difficult to find clones of infected cells using integration site analysis. This observation contrasts with the results presented by Haworth et al [41], who studied the expansion of clones of HIV infected cells using humanized immunodeficient mice. In the humanized mice, which were infected for 12-14 weeks and were not given ART, it would appear that clones of HIV infected cells were larger, and made up a larger fraction of the infected cells, than what we found either in untreated humans or in untreated SIV infected macaques. We suggest that there are at least two possible explanations for the observed differences in the data that we obtained for clones of SIV infected cells in untreated macaques, HIV infected cells in untreated humans, and the data Haworth et al obtained for HIV infected cells in untreated humanized mice. First, the total number of T cells that are targets for infection with HIV or SIV is much smaller in the humanized mice than in it is humans or macaques. Second, in the immunodeficient mice, the human T cells must grow to create a hybrid immune system, which could create bottlenecks and/or impose selective forces on the population of infected cells that does not occur in HIV infected humans or SIV infected macaques. Both of these factors could contribute to having, in the mice, a relatively small number of large clones of HIV infected cells. These effects would give, in the untreated humanized mice, a ratio of newly infected T cells to previously infected T cells that have clonally expanded that is different from what it is found either in untreated humans infected with HIV, or in untreated macaques infected with SIV.

Clonal expansion of HIV infected cells is an important mechanism in the generation and maintenance of the viral reservoir that has made it impossible to cure either HIV-infected humans or SIV-infected macaques with available ART. The results we present here suggest that the greater experimental latitude afforded by nonhuman primate models, including the opportunity to use defined challenge viruses, such as molecularly barcoded viruses that can be used to track distinct viral lineages [42], flexibility in the timing of the initiation, duration, and intensification of ART, as well as intervention with cytokines, immunizations, modulation of cell populations with monoclonal antibodies, and increased opportunities to sample key tissues, should make it possible to address the mechanisms underlying clonal expansion of infected cells, including the roles of homeostatic and antigen driven proliferation. This model shows great promise for facilitating our understanding of the biology of clonally expanded infected cells and for the evaluation of experimental approaches designed to target the expanded clones, which represent a formidable barrier to treatments intended to cure HIV infection.

## Materials and methods

### Infection of human and Rhesus macaque PBMC with SIV in vitro

Rhesus macaque PBMCs from two donors that were not infected with SIV were stimulated with concanavalin A (ConA; 2 μg/ml) and recombinant human IL-2 (100 U/ml) and cultured for three days. Human PBMCs from two donors were stimulated with phytohemagglutinin (PHA; 2.5 μg/ml) and recombinant human IL-2 (30 U/ml) and cultured for four days. Stimulated cells were then washed and inoculated at a nominal MOI of 0.1–0.01 with a stock of SIVmac239 that was generated in transfected 293T cells (purchased from American Type Culture Collection (ATCC), Manassas, VA) and treated with recombinant DNase I (Roche) to eliminate residual plasmid DNA. After 6 hours, input virus was washed out and infected cells were cultured in the presence of IL-2 for 7 days.

### Preparation of uninfected Rhesus macaque PBMC for RNA-Seq analysis

Rhesus macaque PBMCs from two donors were stimulated and cultured as described above in the protocols used to prepare cells for infection with SIV in vitro, but the cells were not inoculated with infectious SIV. PBMC were stimulated with ConA (2 μg/ml) and recombinant human IL-2 (100 U/ml) and cultured for three days. Stimulated cells were then washed and cultured for an additional 7 days in the presence of IL-2 for 7 days.

### RNA-Seq

RNAseq was done using an Illumina TruSeq Stranded total RNA Prep Kit RS-122-2201 following the manufacturer’s protocol. Sequencing was performed on Illumina HiSeq 2500 with TruSeq V4 chemistry with 2x125bp paired end reads. Reads were trimmed for adapters and low-quality bases using Trimmomatic software and aligned with reference Rhesus Macaque Mmul 8.0.1 genome and RefSeq gtf file (UCSC genome downloads) using Star software. Quantification was carried out with RSEM using a transcriptome bam file created by Star.

### Samples from SIV infected rhesus macaques

PBMC and single-cell suspensions of lymph node- and spleen-derived mononuclear cells were prepared as described [43] from samples collected from four Indian origin rhesus macaques (animals DCCN, DCHV, DCT3, and DCJB). The animals were infected and treated as described previously [43]. Briefly, the animals were intravenously inoculated with SIVmac239 and then, at 4 weeks post-infection, started on a combination antiretroviral therapy regimen comprising a co-formulated three drug cocktail of the reverse transcriptase inhibitors tenofovir (TFV) and emtricitabine (FTC) and the integrase strand transfer inhibitor dolutegravir (DTG), plus the protease inhibitor darunavir (DRV). Animals DCCN and DCT3 also received 7–8 infusions of the histone deacetylase inhibitor romidepsin (Istodax). All four animals were housed at the National Institutes of Health (NIH) and cared for in accordance with the Association for the Assessment and Accreditation of Laboratory Animal Care (AAALAC) standards in an AAALAC-accredited facility and all procedures were performed according to protocols approved by the Institutional Animal Care and Use Committee of the National Cancer Institute (Assurance #A4149-01), as described previously [43].

### DNA and RNA preparation

Samples of approximately 100mg or less were homogenized in 1 ml of TriReagent (Molecular Research Center, Inc.) in 2 ml extraction tubes of Lysing Matrix D using FastPrep instrumentation (MP Biomedicals) according to the manufacturer's recommendations. Total RNA and DNA were prepared from the homogenates following manufacturer's recommendations; the alternative, back-extraction method was used for DNA preparation.

### Integration site analysis

Integration site analysis was performed as previously described [1]. Briefly, DNA was isolated from human or macaque PBMC grown in culture, PBMC isolated from the blood of infected macaques, or macaque necropsy tissue samples. Genomic DNA was fragmented by random shearing into 300–500 bp fragments. Linker-mediated nested PCR was performed to amplify the human or macaque genomic regions linked to the SIV sequences from both the 5’ and 3’ LTRs. Paired end-sequencing was carried out using the MiSeq 2x150bp paired end kit (Illumina, San Diego, CA). The sequences of the host-viral junctions and the host DNA breakpoints were determined. The host DNA sequences were then mapped to human genome (hg19) or the Rhesus macaque genome (rheMac8) using BLAT. A stringent filter was used to select the integration sites.

### Identifying clones in pre-therapy samples

Most of the previous analysis of the clonal expansion involved DNA from HIV infected cells in samples taken from individuals who had been on successful ART for years. When exactly the same integration site was found twice (i.e., the identical integration site with exactly two different breakpoints) in samples taken from donors on long-term ART, it was taken as evidence that the cells had clonally expanded. This conclusion was based on the absence of newly infected cells in the donors we were studying. It is formally possible some of the samples from individuals on ART contained infected cells in S, G2, or M that contained two copies of a provirus present only once when the cells were in G1. However, if there are any infected cells that are still dividing after years of successful ART, it is quite likely that they are part of a clone. A similar logic applies to the analysis of samples taken from SIV-infected macaques on ART. The situation is different in an untreated HIV infected human, or an untreated SIV infected macaque, in which there are large numbers of newly infected cells that will live only for a day or two. Both HIV and SIV preferentially infect activated T cells. It is likely that some of the newly infected cells will replicate their DNA but die before they can divide, much less form a clone. As such, finding the same integration site twice in a single sample from an untreated human or macaque is not sufficient to determine that the infected cell was part of an expanded clone. We used two criteria to identify and confirm clones in samples taken from untreated humans and macaques 1) The integration site was isolated three or more times in a single integration site assay. 2) the integration site was seen in two (or more) independent assays, either done with cells taken at the same time, or at different times. For samples taken from humans and macaques on ART, the isolation of the same integration site twice is still considered to be evidence that there is an infected clone of cells in the sample. In samples collected pre-ART, integration sites found with exactly two breakpoints are reported separately from those that could be confirmed as belonging to clones (Table 9).

### Calculation of the likelihood of detecting a clone by integration site analysis relative to the number of cells in the clone in the patient

On average, an HIV-infected individual on therapy has about 1 provirus per 10^3^ CD4+ T cells. Given a total of about 10^10^ CD4+ T cells in blood, and an estimated nearly 10^12^ CD4+ T cells in the whole body, these values imply that there are roughly 10^8^−10^9^ infected cells. Although macaques are smaller, the proviral burden, (the fraction of T cells that are infected) is somewhat larger, so the overall number of infected cells is similar [15, 44]. Some of the infected cells will have clonally expanded and the cells in each infected clone will have a provirus with an identical integration site. We want to know the probability, if we sample the population N times, that we can detect a clone of a particular size. We performed a calculation based on the assumption that we had obtained a total of 100 integration sites, 500 sites, 1000 sites, etc., from an infected individual. Given a population of 10^8^ total infected cells, if we start with the case in which we obtained 1000 integration sites, and if we have a clone that comprises 10^5^ cells, we would expect, on average, to isolate the integration site for the clone once. To detect a clone in a patient or a macaque on ART, we need to see the integration site for the clone of interest at least twice. We can use the Poisson distribution to get the probability of obtaining the same integration site two (or more) times. The probability is the sum of the Poisson values (based on an average of 1) for which the number of positives was greater than or equal to 2, or, more simply, 1 minus the sum of the Poisson expectations for 0 and 1.

### Ethics statement

Peripheral blood mononuclear cells were obtained from healthy donors on an IRB-approved NIH protocol (99-CC-0168). Research blood donors provided written informed consent and blood samples were de-identified prior to distribution (Clinical Trials Number: NCT00001846).

Four Indian-origin rhesus macaques were housed at the National Institutes of Health (NIH) and cared for in accordance with the Association for the Assessment and Accreditation of Laboratory Animal Care (AAALAC) standards in an AAALAC-accredited facility and all procedures were performed according to protocols approved by the Institutional Animal Care and Use Committee of the National Cancer Institute (Protocol AVP-038; Assurance #A4149-01), as described previously [43]. Work involving animals adhered to the standards of the Guide for the Care and Use of Laboratory Animals (National Research Council. 2011. Guide for the care and use of laboratory animals, 8th ed. National Academies Press, Washington, DC) in accordance with the Animal Welfare Act.

Animals were maintained in Animal Biosafety Level 2 housing with a 12:12-hour light:dark cycle, relative humidity 30% to 70%, temperature of 23 to 26°C and all animals were observed twice daily by the veterinary staff. Filtered drinking water was available ad libitum, and a standard commercially formulated nonhuman primate diet was provided thrice daily and supplemented 3–5 times weekly with fresh fruit and/or forage material as part of the environmental enrichment program. Environmental enrichment: Each cage contained a perch, two portable enrichment toys, one hanging toy, and a rotation of additional items (including stainless steel rattles, mirrors, and challenger balls). Additionally, the animals were able to listen to radios during the light phase of their day and were provided with the opportunity to watch full-length movies at least three times weekly. Whole blood was collected from animals sedated with ketamine or via intravenous catheters in conscious animals in restraint chairs. For surgical lymph node extraction, animals were sedated with ketamine and isoflurane inhalation anesthesia, with perioperative administration of a local anesthetic. Pain and distress were relieved by appropriate measures. Animals experiencing more than momentary pain as diagnosed by the veterinarian were treated with appropriate analgesia, including non-steroidal anti-inflammatory drugs, such as ketoprofen, aspirin, and others, and opioids, such as buprenorophine, at the veterinarians discretion. Euthanasia, when appropriate or necessary, was performed in sedated animals using an overdose of sodium pentobarbital.

## Supporting information

S1 Table List of all SIV integration sites with more than one breakpoint. Verified clones present only in on-ART samples are highlighted in yellow. Verified clones in pre-ART samples are highlighted in green.
(PDF)
