# Supplementary material for: Clonal expansion of SIV-infected cells in macaques on antiretroviral therapy is similar to that of HIV-infected cells in humans
Source: PLoS Pathog. 2019 Jul 10;15(7):e1007869. doi: 10.1371/journal.ppat.1007869 (PMC6619828; doi:10.1371/journal.ppat.1007869)
Supplement: S1 Table — Verified clones present only in on-ART samples are highlighted in yellow. Verified clones in pre-ART samples are highlighted in green. (PDF) [file ppat.1007869.s001.pdf]

Table S1 List of all SIV integration sites with more than one breakpoint. Verified clones present only in on-ART samples are highlighted in yellow. Verified clones in pre-ART samples are highlighted in green.

A. DCCN

Integration Site	2 wks PreART	4 wks PreART	PBMC OnART	MesLN OnART	AxLN OnART	Spleen OnART
chr9+1185531		6				
chr17-27611290			7		5	4
chr14+13243179			1		1	3
chr19-37933173			3		1	
chr13-15593747			3			
chr14+605213			3			
chr16-48098270			1	2		
chr19-1158695			1	2		
chr2+121243406			1		1	1
chr20-2178371			1	2		
chr16-2538986			1			1
chr4-78649569			1		1	
chr6-63795004			1			1
chr19-806326				10		
chr2+33598496				4		
chrX-144124016				4		
chr1+135421407				3		
chr16-69927712				3		
chr19-19114474				2		
chr4-26999218				1		1
chr4+34561621				2		
chr6-76382489				2		
chr14-2503865					3	
chr14-9185238					1	2
chr1-145278790					2	
chr14+56222					2	
chr14+9153954					1	1
chr1-101283251						2
chr12-123923740	2					
chr14-19599328	2					
chr14-7117795	2					
chr14+11934160	2					
chr16+75448029	2					
chr13-44121390		2				
**Unique Integration Sites**	**1821**	**294**	**100**	**130**	**101**	**78**

B. DCHV

Integration Site	2 wks PreART	4 wks PreART	PBMC OnART	MesLN OnART	AxLN OnART	Spleen OnART
chr15+81246544			1		2	4
chr1+128886428			3			
chr1-109869086			1	1		
chr19+31813970			1	1		
chr10-61161726				6		
chr19+37213405				5		
chr1-2802025				1		2
chr9-108391356				3		
chr10-49168403				2		
chr14-110841028				1		1
chr14+48297038				1		1
chr16+57185402				1		1
chr19-8480376				1		1
chr19+44959568				2		
chr2-52288218				2		
chr3-36645528				1		1
chr14+7763284					2	2
chr10-78129258					3	
chr10+49607290					1	2
chr16-26460653					3	
chr2+133953512					3	
chr9-95853767					2	1
chr15+1393824					1	1
chr17+29957235					2	
chr19+6447452					1	1
chr2+91716533					1	1
chr4+27092987					1	1
chr13-25985592						3
chr14+9693183						3
chr10+46598758						2
chr14-109836516						2
chr12-123436351	2					
chr16-77108026	2					
chr5-188696727	2					
chr7-159504572	2					
chr5-188696727	2					
chr7-159504572	2					
chr10-82814265	2					
chr19-43584080	2					
**Unique Integration Sites**	**1234**	**82**	**46**	**215**	**103**	**168**

C. DCJB

Integration Sites	2 wks PreART	4 wks PreART	PBMC 12 Months OnART	PBMC 20 Months OnART
chr1-1305541		3		
chr10-63107988		3		
chr16-76193316		3		
chr1+325700			4	2
chr1+325699			1	1
chr16-76131024			1	1
chr18-44412497			2	
chr19-674334			2	
chr10+61547525				2
chr15-17163191				2
chr16-31383332				2
chr16-75416589				2
chr18+1017428				2
chr19-45762004				2
chr19+45266017				2
chr19+50558242				2
chr2-92368983				2
chr8-142491697				2
chr3-185714491	2			
chr4+133898500	2			
chr6+165104357	2			
chr1-128781615		2		
chr1+222902788		2		
chr13-101339855		2		
chr15-1493796		2		
chr16-24763821		2		
chr16-71588975		2		
chr4-34252580		2		
**Unique Integration Sites**	**628**	**746**	**32**	**94**

D. DCT3

Integration Sites	2 wks PreART	4 wks PreART	AxLN OnART	Spleen OnART
chr3+37066653	1			1
chr10-584184		3		
chr2+95052719		3		
chr11-1422670			1	7
chr12+85428795			1	7
chr8-144060762			1	6
chr14+9151331			1	3
chr2-85311071			1	2
chr20+39812193			2	1
chr6+36489420			1	2
chr8-143796222			1	2
chr1-135561547			1	1
chr1+7623893			2	
chr10+78299820			1	1
chr3-85891290			2	
chr3+3792324			1	1
chr9-58363322			1	1
chr16+2041035				4
chr19+42568796				4
chr10+84136583				3
chr14-2794758				3
chr3+37066651				3
chr8-143796225			1	2
chr1+31171834				2
chr14+104088				2
chr14+4052914				2
chr14+6920563				2
chr16+72581183				2
chr19+42112546				2
chr7-159879538				2
chr10-88381834	2			
chr3+37066653	2			
chr7+164426312	2			
chr14-8346012		2		
chr19+791704		2		
chr20-57999121		2		
chr3+45912691		2		
chr6-1010536		2		
chr6-165104362		2		
chr8+142869869		2		
chrX+123962063		2		
**Unique Integrations Sites**	**1291**	**365**	**120**	**174**
